# Supplementary figures and images for: Patient-Derived Orthotopic Xenograft (PDOX) Mouse Models of Primary and Recurrent Meningioma
Source: Cancers (Basel). 2020 Jun 5;12(6):1478. doi: 10.3390/cancers12061478 (PMC7352400; doi:10.3390/cancers12061478)

Supplementary Materials

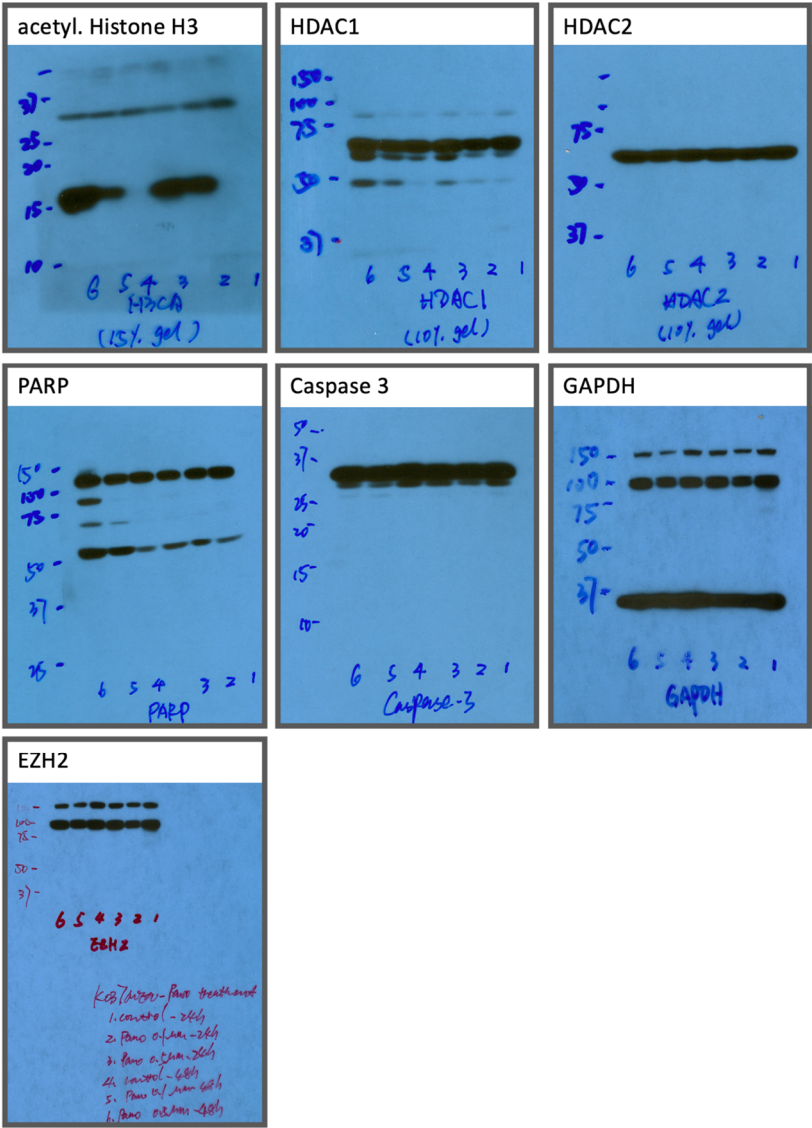

Figure S1. Western blot, unaltered scans.

Supplement: Supplementary file 1 [file cancers-12-01478-s001.pdf]
